# Halophyte Plants as Potential Sources of Anticancer Agents: A Comprehensive Review

**DOI:** 10.3390/pharmaceutics14112406

**Published:** 2022-11-08

**Authors:** Luísa Custodio, Pedro Garcia-Caparros, Catarina Guerreiro Pereira, Pedro Castelo-Branco

**Affiliations:** 1 Centre of Marine Sciences, Faculty of Sciences and Technology, Campus of Gambelas, University of Algarve, 8005-139 Faro, Portugal; 2 Agronomy Department of Superior School Engineering, University of Almeria, 04120 Almería, Spain; 3 Faculty of Medicine and Biomedical Sciences (FMCB), Campus de Gambelas, University of Algarve, 8005-139 Faro, Portugal; 4 Algarve Biomedical Center Research Institute (ABC-RI), 8005-139 Faro, Portugal; 5 Champalimaud Research Program, Champalimaud Center for the Unknown, 1400-038 Lisbon, Portugal

**Keywords:** cancer, natural products, cytotoxic, salt-tolerant plants, antitumoral drugs

## Abstract

Salt-tolerant plants (halophytes) are widely distributed worldwide in several environments such as coastal salt marshes, sand dunes, and inland deserts. To cope with the harsh conditions that characterize those habitats, which include high salinity and radiation levels, such plants have developed morphological and physiological traits, the latter including the synthesis and accumulation of important secondary metabolites such as alkaloids and polyphenols. While essential in maintaining plant homeostasis, these compounds are highly valued in the medical field for the treatment of several human diseases, including cancer. Cancer is one of the most life-threatening disorders worldwide, which accentuates the need to improve current cancer therapies and minimize potential adverse secondary side-effects. In this context, the pharmacological evaluation of natural compounds has attracted growing interest since nature has already provided some important anti-cancer drugs. This review compiles, for the first time, research regarding the anticancer activity of halophytes from different families, including, whenever possible, the bioactive molecules involved in such therapeutical properties along with possible mechanisms of action. The introduction section provides some pertinent information regarding cancer and a summary of the most important characteristics of halophytes. The next section gives information regarding the in vitro and in vivo cytotoxic properties of several halophyte species, grouped by families, including contents in bioactive metabolites and proposed modes of action, if possible. Lastly, the conclusion presents the most relevant metabolites and/or promising species and extracts that could be further explored in anticancer drug research.

## 1. Introduction

Cancer is a group of diseases characterized by malignant neoplasms arising from the abnormal and uncontrolled cell proliferation that invades and destroys the surrounding tissue and, if not controlled, can result in death [1,2]. With an established complex link between cancer and aging and the increased risk factors of an unhealthy lifestyle, cancer is undoubtfully a major health problem in developed and developing countries [3,4,5,6,7]. It is estimated that, by 2040, the number of new cancer cases diagnosed per year will be higher than 27.5 million worldwide, with 16.3 million cancer deaths due to population growth and aging [8]. Globally, lung and breast cancers are the most frequently diagnosed and are the leading causes of cancer-related death in men and women, respectively [9]. Significant advances have been made in cancer research in the last 20 years concerning its general biology, prevention, and treatment [10]. Currently, chemotherapy is one of the most common cancer therapeutic approaches, although secondary effects are known and include a negative impact on the immune system, and the effectiveness of this therapeutic approach can be limited by drug resistance [11,12]. Therefore, new anticancer drugs and therapies that concomitantly minimize its harmful side-effects are still in urgent demand.

In the war against cancer, the role of natural compounds has become of crucial interest, and the scientific community has placed great effort in identifying novel sources of antitumoral molecules among medicinal plants and, more recently, marine organisms [10,13]. The pharmacological evaluation of natural compounds has rendered some important anticancer drugs such as paclitaxel and its derivatives from the bark of the Pacific yew tree (*Taxus brevifolia* Nutt, family: Taxaceae), or vincristine and vinblastine from Madagascar periwinkle (*Catharanthus roseus* (L.) G. Don, family: Apocynaceae) [14]. However, in addition to glycophyte plants and some marine species, other organisms including halophytes contain metabolites with potential use as anticancer drugs.

Although the definition of halophytes has yet to reach consensus, some authors define them as plants completing their life cycle in environments with a salt concentration of around 200 mM of sodium chloride (NaCl) (20 dS m^−1^) or more [15,16,17]. They represent nearly 1% of the global flora diversity and inhabit different ecosystems such as beaches, rocky shores, saltmarshes, estuaries, and inland deserts [17,18,19]. Such areas are characterized by stressful abiotic conditions, (e.g., high salinity, radiation levels, and drought), which trigger the production and accumulation of reactive oxygen species (ROS) in the plants, leading to cellular and tissue damage, metabolic disorders, and senescence. Halophytes are equipped with strong antioxidant defense systems to counteract the negative effects of ROS, including antioxidant enzymatic mechanisms and the synthesis of secondary metabolites, such as phenolic compounds, saponins, and alkaloids [18,20]. These molecules have a crucial protective role in the plant and exhibit relevant bioactivities, including antioxidant, anti-inflammatory, and antitumoral, which are linked to beneficial therapeutic properties and could help explain the use of some halophytes in traditional medicine and as food [18,21,22].

There are several studies focused on the anticancer properties of a high number of halophytic species, but this information is scattered in the literature. Therefore, our aim was to compile and summarize existing information regarding the anticancer activity of halophytes, the molecules involved in such properties, and the possible mechanisms of action (Table 1, Table 2, Table 3 and Table 4). The Web of Science database and Google Scholar (as a search engine) were consulted to retrieve the most updated articles on the topic under investigation. The keywords “salt-tolerant plants” and “halophytes” were used alone or in combination with, for example, “anticarcinogenic”, “antitumoral”, or “anticancer”. Only English articles having full text were considered. The contribution was distributed according to the different families and species, which were previously checked in the eHALOPH database [23]. The correspondence between the name of the cell lines referred in the text and the type of cancer can be found in Table 1, together with a list of halophyte species, organs and extracts tested, cell lines/types of cancer assessed, and obtained half-maximal inhibitory concentrations (IC_50_) values and proposed mechanisms of action, when provided by the authors. However, at the end of the study, a summary is presented referring only to the results obeying the criteria of cytotoxic activity for crude extracts (IC_50_ < 30 μg/mL; Table 3) and isolated compounds (IC_50_ ≤ 10 μM; Table 4) considered relevant to proceed for antitumoral applications, as established by the American National Cancer Institute (NCI) [24] and the National Institutes of Health (NIH) [25], respectively.

## 2. Anticancer Activity of Halophytes

Halophytes are a valuable source of bioactive molecules for the prevention and treatment of human chronic diseases, including cancer [26]. In this review, information is presented by family, namely, *Acanthaceae*, *Aizoaceae*, *Amaranthaceae*, *Apiaceae*, *Asteraceae*, *Brassicaceae*, *Convolvulaceae*, *Cymodoceaceae*, *Cyperaceae*, *Fabaceae*, *Juncaceae*, *Malvaceae*, *Myrtaceae*, *Plantaginaceae*, *Plumbaginaceae*, *Poaceae*, *Portulacaceae*, *Rhizophoraceae*, *Solanaceae*, and *Tamaricaceae*. Data regarding the anticancer activity of different halophyte species, including organs and extracts tested, cell lines/types of cancer assessed, and obtained IC_50_ values, are summarized in Table 1. Table 2 contains detected molecules in the active extracts from halophyte species with anticancer properties, while Table 3 summarizes information on extracts/fractions of selected species that could be further explored in the search for new drug-leads, as established by the American National Cancer Institute (NCI) [24], including cytotoxic activity and chemical composition of the extracts (when possible), cell lines tested, obtained IC_50_ values, and proposed mechanisms of action. Lastly, data related to the cytotoxic activity of selected isolated compounds, according to the criteria of the National Institutes of Health (NIH) [25], are presented on Table 4.

### 2.1. Acanthaceae Family

*Acanthaceae* currently comprises 192 genera and 5504 species growing in Indonesia, Malaysia, Africa, Brazil, and Central America [27,28]. The presence of bioactive metabolites such as alkaloids, phenols, terpenoids, tannins, quinones, cardiac glycosides, saponins, carbohydrates, and flavonoids, highlights the medicinal relevance of this family in the treatment of several diseases, including cancer [29]. This review focuses on the anticancer activity of the halophytic genera *Acanthus* and *Avicennia*.

*Acanthus ilicifolius* L. (holly leaved acanthus) is a mangrove shrub rich in triterpenes, alkaloids, and flavonoids, traditionally used in Chinese and Indian medicine against asthma, headache, and skin diseases [30]. An ethanolic leaf extract from *A. ilicifolius* significantly reduced the viability of HepG2 cells in a dose-dependent manner (92% of cytotoxicity at 100 μg/mL) via apoptosis induction potentially associated with DNA damage [31] (Table 1). A water root extract from *A. ilicifolius* reduced the viability of HepG2 cells, as observed in the 3-(4, 5-dimethylthiazol-2-yl)-2, 5-diphenyl tetrazolium bromide (MTT) assay (IC_50_ value of 39.76 μg/mL after 48 h of incubation) [32], via apoptosis induction related to DNA damage (Table 1). Ethanol extracts from leaves and roots of holly leaved acanthus were also able to reduce the viability of MCF7 cells (breast carcinoma), with IC_50_ values of 24.22 and 29.20 μg/mL for leaf and root extracts, and of PA1 cells (ovarian carcinoma), with IC_50_ values of 15.74 μg/mL and 20.00 μg/mL for leaf and root, respectively (Table 1) [33]. The chemical profile of the active extracts was not established. *Acanthus ebracteatus* Vahl. (holly mangrove), is traditionally used in Thai medicine as an anticancer agent [34]. A protein hydrolysate (<3 kDa) from the aerial parts of *A. ebracteatus*, reduced the viability of A431 cells, with an IC_50_ value of 425.9 ng protein/mL after 24 h of incubation (Table 1). Partially purified peptides were obtained from this hydrolysate and resulted in the synergistic effects against cell viability of A431 cells, via apoptosis induction [34].

*Avicennia* comprises mangroves distributed in the Indo-Western Pacific and Atlantic eastern Pacific areas. Some *Avicennia* species are effective against cancer due to the presence of different classes of molecules, including alkaloids, phenols, flavonoids, tannins, iridoid glucosides, and terpenoids [35]. A methanol leaf extract from *Avicennia alba* Blume significantly reduced the viability of MCF7 and HeLa cells, evaluated by the MTT assay, with IC_50_ values of 57.02 and 44.30 µg/mL, respectively, after 48 h of incubation [36] (Table 1). After treatment with the leaf extract of *A. alba* both cell lines exhibited morphological modifications, i.e., a reduction in cell size and cell detachment. The extract was analyzed using gas chromatography–mass spectrometry (GC–MS) allowing for the identification of 12 compounds, the most abundant ones being catechol borane (11%), neophytadiene (17%), and hexadecanoic acid (29%) (Table 2), but their cytotoxicity toward the tested cell lines was not established [36]. Moreover, since GC–MS mainly allows for the detection of lipophilic molecules, one cannot exclude the possibility that hydrophilic compounds with cytotoxic properties may be present in the methanol leaf extract but were not detected with the chromatographic system used. A chloroform/methanol extract from leaves of *A. alba* reduced the viability of WiDr cells (IC_50_ = 173.78 µg/mL after 24 h of incubation) using doxorubicin as the positive control and induced cell arrest in the G0–G1 phase, resulting in apoptosis induction [37] (Table 1). 

*Avicennia marina* (Forssk.) Vierh, one of the most abundant and common mangrove species, is used in traditional medicine for the treatment of different diseases, including rheumatism and smallpox, and it contains different molecules with therapeutic properties, such as luteolin 7-O-methylether and chrysoeriol 7-O-glucoside [38]. An ethyl acetate extract of leaves and stems of *A. marina* reduced the viability of MCF7 cells (MTT assay), with values of cell growth inhibition of 65% and 75% after treatment for 48 h with the extract at 100 and 200 µg/mL, respectively [39] (Table 1). When applied at the concentration of 200 µg/mL the extract induced apoptosis in a concentration-dependent manner through ROS production and disruption of the mitochondrial membrane potential (Δψm); however, no PARP-1 cleavage was detected, and a significant decrease in caspase-7 protein levels was observed. A significant level of autophagy was also detected at the same concentration [39]. A methanol extract of the same species reduced the viability of HeLa cells in a concentration-dependent manner (tested concentrations: 50, 100, 250, 500, and 1000 µg/mL) with an IC_50_ value of 107 µg/mL [40] (Table 1). The chemical profile of the extracts was not established by the authors and, therefore, the bioactive compounds were not identified. In another study targeting the same species [38], water, ethanol, methanol, and ethyl acetate extracts from leaves and seeds were tested toward different human cancer cell lines, namely, breast (AU565, MDA-MB-231, and BT483), liver (HepG2 and Huh7), and NIH3T3 cells. The ethyl acetate extract from leaves had the highest cytotoxicity, especially toward cancer cells, which was attributed by the authors to its highest phenol content. The ethyl acetate extract suppressed xenograft MDA-MB-231 tumor growth in nude mice, and induced apoptosis in cancer cells through apoptosis inhibition and inhibition of cell migration. This extract was further fractionated by column chromatography, and resulting fractions were retested for anticancer properties. The most active factions, F2-5, F3-2-9, and F3-2-10, had IC_50_ values lower than 20 μg/mL, and they were profiled by ^1^H-NMR and ^13^C-NMR, allowing for the identification of the flavonoids luteolin and quercetin standards, and avicennones D and E. The antiproliferative activity of the flavonoids was confirmed [38]. Additional studies about the anticarcinogenic activity of other halophytic species of the *Acanthaceae* family can be found in Table 1.

### 2.2. Aizoaceae Family

*Aizoaceae* contains mostly succulent plants with 1910 species currently recognized, distributed across 125 genera [41,42]. The *Mesembryanthemum* genus, for example, is used in traditional medicine to treat several diseases such as hepatic conditions and diabetes, and it contains several bioactive compounds, including flavonoids and catechins [43]. An ethanol crude extract from leaves of *Mesembryanthemum crystallinum* L. (ice plant), and obtained hexane, ethyl acetate, butanol, and water fractions were tested for their effect on the growth of HCT116 cells [44] (Table 1). The application of the ethyl acetate and butanol fractions resulted in a dose-dependent inhibition of cell growth (at 125, 250, and 500 μg/mL, applied for 24 and 48 h), as observed in the MTT assay, a reduction in the levels of intracellular ROS (at 250 and 500 μg/mL, using 2′,7′-dichlorofluorescin-diacetate), and apoptosis induction (at 250 and 500 μg/mL, during 48 h). Treatment with the butanol fraction also resulted in cell-cycle arrest in the G2/M phase [44]. Samples were evaluated for total phenolic content (TPC), using the Folin–Ciocalteau (F–C) assay, and it was observed that the butanol fraction had the highest TPC (5.4 mg gallic acid equivalent/g) [44], suggesting that phenolics may be related to the detected cytotoxicity. However, no attempt was made by the authors to identify the active molecules present in this sample.

The application of ethanol, methanol, acetone, hexane, and diethyl-ether extracts from whole plants of *Sesuvium portulacastrum* L. (sea purslane) decreased the viability of MDA-MB-231, IMR32, and HCT116 cell lines, as assessed by MTT after 24 h of incubation, using doxorubicin as the standard [45]. The hexane extract had the highest cytotoxic activity with IC_50_ values of 942.07, 703.40, and 407.87 µg/mL for MDA-MDB-231, IMR32, and HCT116 cell lines (Table 1). Despite the considerably high IC_50_ values obtained, the possible mechanisms of action of the extracts were evaluated using phase contrast-inverted microscope observation and propidium iodide staining, which revealed the presence of typical features of apoptotic cells, including nuclear condensation, cell shrinkage, and the presence of apoptotic bodies [45] (Table 1). No attempt was made by the authors to unravel the chemical composition of the extracts.

### 2.3. Amaranthaceae Family

*Amaranthaceae* currently comprises 2398 species grouped in 182 genera [46], which can be found in a wide range of habitats from arid and semiarid regions to saline environments and subtropical areas; it contains flavonoids, phenolic acids, terpenes, and triterpene saponins, and it displays important pharmacological properties such as anticarcinogenic [47,48].

*Arthrocnemum* sp. is a small genus of succulent halophytes present in coastal marshes of South Africa, Asia, Mediterranean, Europe, and Northern America, and it contains several compounds such as phenols, flavonoids, and tannins with described therapeutic properties, including antibiotic, hypoglycemic, and antitumoral [49,50]. The application of an 80% methanol extract from shoots of *Arthrocnemum indicum* (Willd.) Moq. significantly reduced the viability of Caco-2 cells, as observed by the MTT assay [50] (Table 1). Fluorescence microscopy observation through DAPI (4,6-diamidino-2-phenylindole) staining showed that cells exhibited a decline of DNA synthesis, while flow cytometry allowed for the observation of cell-cycle arrest at the G2/M phase after 72 h of exposure to the extract at the concentration of 100 µg/mL [51] (Table 1). The extract was profiled by liquid chromatography/electrospray ionization time-of-flight mass spectrometry (LC/ESI-TOF-MS), and high levels of phenolic compounds were detected such as gallic acid, cyanidin, chrysoeriol, quercetin, catechol, syringic acid, and luteolin (Table 2), although their cytotoxicity toward Caco-2 cells was not established [51]. However, it is known that some of the detected compounds exhibit antitumoral properties. For example, chrysoeriol was identified as a selective inhibitor of the PI3K–AKT–mTOR pathway and, therefore, linked with cell cycle regulatory effects; it also reduced the proliferation of human multiple myeloma cells (RPMI 8226 and KM3, with IC50 values of 26 and 35 µmol/L at 48 h) but not of peripheral blood mononuclear cells (PBMNCs) [52].

Several *Atriplex* species are halophytic [53]. An ethanol extract from leaves of *Atriplex halimus* L. (sea orache) significantly reduced the viability of HepG2 cells (IC_50_ = 54.86 µg/mL after 24 h of incubation) (Table 1) via apoptosis induction linked to the expression of TP53, BCL2, and BAX genes [54] (Table 1). No information was provided by the authors regarding the chemical components of such extract, but it is known that *A. halimus* is characterized by the presence of different bioactive compounds, including syringetin derivatives and the flavonol glycosides atriplexoside A [3′-*O*-methylquercetin-4′-*O*-β-apiofuranoside-3-*O*-(6′′-*O*-α-rhamnopyranosyl-β glucospyranoside) and atriplexoside B [3′-*O*-methylquercetin-4′-*O*-(5′′-*O*-β-xylopyranosyl-β-apiofuranoside)-3-*O*-(6′′-*O*-α-rhamnopyranosyl-β-glucopyranoside)], which may be related to the detected cytotoxic activity [55,56,57,58,59,60].

*Chenopodium* contains weedy herbs native to Asia, Europe, and America, rich in phenolics, saponins, and triterpenoids, conferring them therapeutic properties such as laxative, analgesic, and anticarcinogenic [61]. The edible species *Chenopodium album* L. (lamb’s quarters) is valued in traditional medicine for its anticancer properties [62]. A petroleum ether extract of branches and leaves from *C. album* reduced the viability of A549 cells in a dose-dependent manner, showing an IC_50_ of 33.31 μg/mL using gemcitabine as a positive control, which was linked to cell-cycle arrest at the G1 phase [63] (Table 1). Essential oil from whole plants of *Chenopodium ambrosioides* L. (Mexican tea) decreased the viability of MCF7 cells by inducing DNA fragmentation (IC_50_ values of 18.75, 9.45, and 10.50 μg/mL at 6, 24, and 48 h, respectively) when compared to the control treatment [64] (Table 1). In another study, an essential oil of the same species also displayed a significant cytotoxic activity toward RAJI cells, with an IC_50_ value of 1.0 μg/mL, which was probably related to the high levels of ascaridole, detected using GC with flame ionization detection (GC-FID), GC–MS, and proton nuclear magnetic resonance (1H-NMR) [65] (Table 1 and Table 2). Ascaridol is a monoterpene with a strong in vitro capacity to decrease the growth of different tumor cell lines and is, therefore, considered as a strong candidate for the treatment of cancer [65]. *Chenopodium quinoa* Willd is a pseudocereal originated from the Andes of South America with recognized nutritional and functional properties that rendered the species the status of “functional food” [66]. Polysaccharides were extracted from quinoa seeds with petroleum ether and purified by column chromatography, revealing mainly galacturonic acid and glucose monosaccharides. This polysaccharide fraction was applied toward cancer (SMMC 7721 and MCF7) and “normal” (L02 and MCF 10A) cell lines, for 24 and 48 h, allowing for IC_50_ values ranging from 53.4 to 121.4 μg/mL, without affecting “normal” cell viability [66] (Table 1).

*Salicornia* species (glasswort, sea asparagus, or samphire) are edible succulent halophytes highly appreciated in gourmet cuisine due to their organoleptic properties [67]. The ethyl acetate and methanol leaf extracts from *Salicornia europaea* L. reduced the viability of MCF7 cells with IC_50_ values of 97.9 and 117.1 μg/mL for the ethyl acetate and methanol extracts, respectively, after 24 h of incubation [68]. A qualitative analysis of the extracts identified tannins and saponins, which may be associated with the in vitro antitumor capacity. Moreover, a GC–MS analysis showed the presence of 32 and 29 compounds in corresponding ethyl acetate and methanol extracts, respectively, which were not assessed individually against the cancer cells [68]. Tannins are phenolic compounds with a high molecular weight, while saponins contain a steroidal or triterpenoid aglycone and one or more sugar chains [69]. Both groups of compounds exhibit antitumoral properties; for example, saponins generally decrease cellular proliferation by binding to cholesterol structures on cell membranes, forming pores and holes by binding to specific receptors followed by the induction of apoptosis, while tannins can hamper cancer cell proliferation through antioxidant processes and apoptosis induction [70,71]. However, the presented results must be analyzed with caution, since the phytochemical analysis reported by the authors only targets lipophilic compounds, without unraveling the possible cytotoxic molecules present in the methanol (polar) extract.

*Suaeda* comprises around 80 to 100 succulent species distributed in semideserts, deserts, and seashores. These species are usually edible and contain several bioactive components, such as phenolics, flavonoids, and terpenoids, conferring them with bioactive properties, including anticarcinogenic [72,73]. A hexane extract from leaves of *Suaeda ruticose* (L.) Forssk. (shrubby seablight) reduced the viability of HCT116, HepG2, and MCF7 cell lines, being more effective toward HCT116 cells (IC_50_ = 17.2 µg/mL) [74] (Table 1 and Table 4). The cytotoxicity of the extract was tested using the sulforodamine B (SRB) assays for 72 h using doxorubicin as a positive control with IC_50_ values of approximately 0.5 µg/mL. This extract caused cell-cycle arrest at the G0–G1 phase and induced apoptosis, especially in HCT116 cells that exhibited chromatin condensation and membrane blebbing (Table 4). The extract was profiled using liquid chromatography coupled to electrospray ionization quadrupole time-of-flight mass spectrometry (LC–ESI-QTOF-MS/MS) and several molecules were identified, including monoterpenes (dihydrojasmone, jasmolone, terpinene-4-ol), diterpenes (pimaric acid, steviol, and momilactone B), and phenolics (quercinol, zingerone, zingerol, and neovaflan) (Table 4), but its contribution to the detected cytotoxicity was not directly established [74]. However, there are several reports on the antitumoral properties of phenolic compounds [56,57,58,59]. Specifically, zingerone reduced the viability of HCT116 cells through ROS-mediated apoptosis in colon cancer cells [75]. Monoterpenes and diterpenes have reported cytotoxic properties [76,77]. For instance, dihydrojasmone was identified as one of the active compounds toward HeLa and NIH/3T3 cell lines, in a methanol extract from *Rumex hastatus* D. Don [78], while steviol significantly reduced the viability of MCF7 cells via apoptosis induction [79]. A dichloromethane extract from the shoots of *S. fruticosa* significantly reduced the viability of A549 (IC_50_ = 49 µg/mL), DLD1 (IC_50_ = 10 µg/mL), Caco-2 (IC_50_ = 140 µg/mL), and HT-29 cells (IC_50_ = 12 µg/mL), which was assessed using the resazurin reduction test after 48 h of incubation (Table 1 and Table 4). Nevertheless, the bioactive compounds present in the chemical profile of the extracts were not identified [80]. Additional studies on the anticarcinogenic activity of other halophytic species of this family are summarized in Table 1.

### 2.4. Apiaceae Family

*Apiaceae* currently comprises 3916 species distributed across 457 genera dispersed worldwide [81], and it is characterized by the presence of polyacetylenes displaying important bioactivities, including anticarcinogenic [82]. In this family, there are two genera of halophytic species, *Eryngium* sp. and *Crithmum* sp., with described cytotoxic properties against human cancer cells.

*Eryngium* includes approximately 250 species present in Eurasia, North Africa, North and South America, and Australia. *Eryngium* species are frequently used as ornamentals or as a food source and are valued in traditional medicine as a diuretic, to treat diarrhea, headaches, and digestive problems. These properties are potentially associated with the presence of terpenoids, triterpenoids, polyacetylenes, flavonoids, and coumarins [83]. The cytotoxic effects of aqueous extracts from shoots and roots of *Eryngium maritimum* L. were assessed against human cancer lines (HepG2, HEP-2, and U138MG) and on a “normal” cell line (Vero), using the MTT assay after 24 h of incubation [70] (Table 1). The root extracts were overall more cytotoxic, and the IC_50_ values ranged from 30.3 to 50 µg/mL. However, a strong reduction in cellular viability was also observed in the “normal” cell line, which suggest no selectivity toward cancer cells [70] (Table 1). Although the phytochemical profile of the extracts was not established by the authors, other reports identified several cytotoxic molecules in *E. maritimum*, including saponins, which create pores and perforations on the cellular membranes by binding to specific receptors, followed by apoptosis induction by, for example, stimulation of the cytochrome c-caspase 9-caspase 3-pathways [70,71].

*Crithmum maritimum* L. (sea fennel) is traditionally used as a cooking ingredient and in folk medicine for its stimulating and diuretic effects [84]. An ethyl acetate extract from the whole sea fennel plant reduced the proliferation of Huh7 and HepG2 cell lines, by interfering with the cell cycle, specifically on the shift of phases with increasing number of cells in the G0/G1 phase after 24 h of incubation and in the G2/M phase after 48 h [85]. The extract induced an increase in necrotic and apoptotic cancer cells, assessed by cytofluorimetric analysis although the values of IC_50_, but the chemical profile of the extract was not assessed [85] (Table 1).

### 2.5. Asteraceae Family

*Asteraceae* currently contains 1733 genera and 35,988 species [86], including several used as ornamentals (e.g., *Calendula* and *Chrysanthemum*) or for food (e.g., *Helianthus annuus*) and medicinal (e.g., *Artemisia* sp. and *Echinacea* sp.) purposes [87]. *Achillea millefolium* L. (yarrow) is an aromatic perennial herb with traditional medicinal uses such as wound healing and anti-inflammatory activity, and it contains different classes of bioactive compounds, including flavonoids and terpenoids [88]. A methanol extract from yarrow shoots was applied toward prostate cancer (DU145) and “normal” skin (HFFF2) cells, at different concentrations (20, 100, 500, 1000, and 2000 µg/mL), alone or in combination with bleomycin, an anticancer agent. The yarrow extract was not toxic toward “normal” cells, but had significantly enhanced cytotoxicity induced by bleomycin showing 60% and 49% survival rate at doses of 1000 and 2000 µg/mL, respectively, which may indicate that this extract contains molecules able to improve the effectiveness of bleomycin, while minimizing negative side-effects caused by toxicity toward “normal” cells. [88] (Table 1). The extract was not profiled for chemical components, but there are reports of the presence of several cytotoxic compounds in yarrow, such as achillinin A (guaianolide) and casticin (flavonoid) [88,89]. In another work, petroleum ether, ethyl acetate, methanol, and water extracts were prepared from aerial organs of yarrow and tested for toxicity, using the MTT assay, on human cancer cell lines [90] (Table 1). The strongest cytotoxic effect was observed after application of the ethyl acetate extract on HeLa (IC_50_ = 0.58 μg/mL) and K562 cells (IC_50_ = 0.73 μg/mL), followed by the water (MCF-7, IC_50_ = 0.87 μg/mL) and the petroleum ether extract (K562, IC_50_ = 0.87 μg/mL) (Table 1 and Table 4) [90]. The cytotoxic activity of these extracts may be associated with its contents in phenolic acids, such as apigenin and chlorogenic, *p*-coumaric, and rosmarinic acids (Table 4), which showed the capacity to block oncogenic pathways due to the activation of caspases [91]. A methanol extract from aerial parts of *Limbarda crithmoides* (L.) Dumort (commonly known as *Inula crithmoides* L.) was able to decrease the viability of acute myeloid leukemia cells (OCI-AML3) when applied at 100 and 200 μg/mL, for 24 h [92] (Table 1). This extract was submitted to a solvent–solvent partitioning, affording *n*-hexane, dichloromethane, and aqueous methanol-soluble fractions. The hexane and dichloromethane fractions exhibited a strong cytotoxicity toward OCI-AML3 cells at concentrations of 15 or 10 µg/mL, which was ascribed to an increase in apoptotic cells, especially in the G0/G1 phase by the mitochondria-dependent pathway. The hexane extract was then further fractionated, leading to the isolation of two molecules, which were identified by NMR as the thymol derivatives 10-acetoxy-8,9-epoxythymol tiglate and 10-acetoxy-9Z-chloro-8,9-dehydrothymol, with the latter being the most active, causing a decrease in cell viability at 1.25 µg/mL associated with apoptosis induction [92].

### 2.6. Brassicaceae Family

*Brassicaceae* is one of the largest dicotyledon family of flowering plants, including model species and commercial crops, with 341 genera and 3921 species recognized at the moment [93,94], but few studies have described the potential anticarcinogenic properties of halophytes belonging to this family. One example is the annual halophyte *Cakile maritima* Scop. (sea rocket), which is confined to maritime strandlines of sand and has agronomic (oilseed and phytoremediation) and medicinal (diuretic, antiscorbutic, and purgative) properties [95]. Hexane, ethyl acetate, and methanol fractions were obtained from a methanol extract prepared from aerial organs of sea rocket and tested for antiproliferative properties on Caco-2 and HeLa carcinoma cells [96] (Table 1). The hexane fraction significantly reduced the viability of Caco-2 and HeLa cells, with IC_50_ values of 12 and 126 μg/mL after 24 h of incubation; respectively. Cisplatin was used as the positive control with values of 69 μg/mL (Caco-2) and 85 μg/mL (HeLa). The extract was profiled using GC–MS, and the major molecules identified were, by area, 2-hydroxy-1,8-cineole, decane, and limonene, which may contribute to the detected antiproliferative activity [96] (Table 4). For instance, limonene has been reported with anticarcinogenic activity in HepG2 cells due to apoptosis induction [97].

### 2.7. Convolvulaceae Family

*Convolvulaceae* currently contains 50 genera and 1952 species widely distributed in tropical and temperate regions of the world [98,99]. *Calystegia soldanella* (L.) R. Br. ex Roem. & Schult. (shore bindweed) is a perennial edible herb commonly found in coastal sand dunes and foredunes of South Korea, East Asia, Europe, and the Pacific. Shore bindweed is traditionally used for the treatment of, for example, rheumatic arthritis and scurvy, and it displays relevant biological properties, including anticancer [100]. An 85% aqueous fraction from combined methylene chloride and methanol crude extracts obtained from whole plants of shore bindweed significantly decreased HepG2 cellular viability after 24 h of incubation, in a concentration-dependent manner, via cell-cycle arrest at the G0–G1 and S phases and apoptosis induction [100]. In another study, a methanol crude extract from whole plants of the same species exhibited a potent cytotoxic activity toward A549 cells (human lung cancer) and Col2 cells (human colon cancer), with IC_50_ values of 8.0 µg/mL and 27.4 µg/mL, respectively [101]. A hydroalcoholic extract from shoots of *Cressa cretica* L. decreased the viability of HepG2 cells (IC_50_ value = 2300 µg/mL after 72 h of incubation), by increasing the expression of the proapoptotic protein BAX in detriment to antiapoptotic proteins (BCL2) [102].

### 2.8. Cymodoceaceae Family

This family presently contains six genera and 17 species of seagrasses [103,104]. A water leaf extract from *Cymodocea rotundata* Ehrenb Hempr. ex Aschers. was supplemented with silver nitrate (AgNO_3_, 1 M), to produce silver nanoparticles (AgNPs). AgNPs have several medical applications, including coating of medical devices and wound dressings, and they exhibit cytotoxic activity [105]. The obtained AgNPs exhibited high cytotoxicity toward MG63 cells with an IC_50_ value of 25.31 µg/mL after 48 h of incubation [105]. AgNPs were also produced by combining a water leaf extract of *Cymodocea serrulata* (R. Br.) Aschers. & Magnus and AgNO_3_ (1 M), and they reduced the viability of A549 cells in a direct dose–response manner (IC_50_ = 100 µg/mL after 24 h of incubation). The higher cytotoxicity of the AgNPs was ascribed to the easy permeability to the cellular barriers and their high affinity to biological macromolecules, as well as their capacity to release ROS [106]. No attempt was made to identify the active molecules present in those samples [105,106]. A hydroethanolic extract from shoots of the same species inhibited the proliferation of HepG2 cells (IC_50_ value of 82.92 µg/mL after 24 h of incubation with camptothecin as a positive control with an IC_50_ value of 8 µg/mL) [107]. The extract had a high concentration of tannins, flavonoids, and terpenoids, with described antitumoral properties [108], but no attempt was made by the authors to identify the possible bioactive molecules [107].

### 2.9. Cyperaceae Family

*Cyperaceae* currently comprises 92 genera and 5888 species with a cosmopolitan distribution [109,110]. *Cyperus rotundus* L. (nut grass) is a perennial halophyte species traditionally used in the treatment of several pathologies, such as stomach disorders [111]. Methanol, ethanol, and water extracts from nut grass rhizomes were tested toward MDA-MB-231 cells, for 24 h [112] (Table 1). The highest reduction in cell viability was obtained with the ethanol extract, with an IC_50_ value of 225 µg/mL, through apoptosis induction via upregulation of the death receptor 4 (DR4), DR5, and proapoptotic BAX, and downregulation of antiapoptotic survivin and BCL2 [112]. The active extract was not chemically profiled, but it is known that the rhizomes of nut grass are rich in several bioactive molecules, including flavonoids, tannins, and sesquiterpenes [113], with reported cytotoxic activity against cancer cell lines.

### 2.10. Fabaceae Family

This family currently comprises 778 genera and 22,356 species [114] and includes five genera of halophytic species, namely, *Alhagi*, *Glycyrrhiza*, *Melilotus*, *Prosopis*, and *Sesbania*, with described anticarcinogenic properties [115].

The genus *Alhagi* is distributed throughout Asia, Australia, and Europe and used traditionally for the treatment of, for example, gastroenteritis, ulcers, and rheumatoid arthritis [116]. *Alhagi maurorum* Medik (camelthorn) is used for its anti-inflammatory properties, which are ascribed to the presence of the triterpenoid lupeol [117]. In fact, lupeol was isolated from a methanol extract of camelthorn aerial parts, and it decreased the cellular viability of MCF7 and MDA-MB-231 cell lines, with one-fourth of IC_50_ values >100 µg/mL (Table 1) [118]. The cytotoxic activity of lupeol was lower than that observed with its epoxide form, and it was related to the increase in mRNA expression levels of apoptosis-related genes (TP53, caspase-3 and BAX) and decrease in BCL2 gene expression [118].

*Glycyrrhiza* (liquorice) contains legumes endogenous to Asia and southern Europe with reported anti-inflammatory and antiviral properties [119]. There are two *Glycyrrhiza* halophytic species with anticarcinogenic activity: *Glycyrrhiza glabra* L. and *G. uralensis* Fisch. *Glycyrrhiza glabra* is rich in phenolics, tannins, and especially glycyrrhizin, a triterpenoid saponin [120]. Glycyrrhizin reduced the proliferation of HeLa cells at 320 µM after 24 and 48 h of incubation, via apoptosis, through mitochondrial depolarization. Moreover, nuclear condensation, cell membrane lysis, and disintegration of organelles were observed in treated cells through phase-contrast microscopy [121]. The cytotoxic potential of the roots’ methanol extracts from *G. glabra*, collected in nine different areas from Italy, Turkey, Syria, Russia, Afghanistan, and Uzbekistan, was assessed against “normal” (HaCaT) and cancer cell lines (A549 and HepG2) using the MTT assay, after 24 h of incubation [122]. Results disclosed variable cytotoxicity levels depending on the samples’ collection location and season, potentially related to the influence of climatic conditions on the chemical composition of the plants. Only one sample from Afghanistan was active toward HepG2 cells (IC_50_ of 248.5 µg/mL), while four extracts (from Italy, Afghanistan, and Syria) were cytotoxic toward A549 cells (IC_50_ between 189.1 and 238.9 µg/mL) [122] (Table 1). However, samples were also cytotoxic toward “normal” cells (HaCat) (Table 1). An ethanol root extract of *G. glabra* significantly reduced the proliferation of HT29 cells, at 200 µg/mL after 24, 48, and 72 h of incubation as detected by the MTT assay [123]. Polymerase chain reaction (PCR) studies revealed a downregulation of heat-shock protein 90 (HS90) gene expression that can be related to the reduction in cellular viability, since the HSP90 prevents tumor cells from undergoing apoptotic death; therefore, its blocking could assist active antitumor effects [124]. A reduction in HeLa cell viability above 80% was reported after application of an aqueous ethanol extract of *G. uralensis* rhizomes at the concentration of 1.84 mg/mL, but the determination of the IC_50_ value was not reported [125]. Several cytotoxic compounds were identified in that extract using UPLC–ESI-Q-TOF, including isoquercitrin, 4′demethylpodophyllotoxin glucoside, and podophyllotoxin, all with described cytotoxic properties [125] (Table 2).

*Melilotus* contains 19 species of annual herbs widely distributed in North Africa and Eurasia [126]. *Melilotus indicus* L. All. (sweet clover), the only described halophyte within this genus, is used in traditional medicine as an analgesic, and it has reported cytotoxic activity [127,128]. A methanol extract from the aerial parts of sweet clover significantly decreased cell proliferation of HepG2 (IC_50_ = 16.60 µg/mL) and SNU-182 cells (IC_50_ = 13.21 µg/mL) after 24 h of incubation, using staurosporine as a positive control (Table 1) [127,128]. The extract was less cytotoxic toward L-02 cells (human “normal” hepatic) (IC_50_ = 90.9 µg/mL) (Table 1) [127,128]. The application of the extract resulted in an increase in the number of apoptotic cells and loss of mitochondrial membrane potential (Δψm) [128] (Table 1). The chemical composition of the extract was not established by the authors.

*Prosopis* comprises 44 species, mainly small trees distributed in dry lands of America, Africa, and Asia. Several *Prosopis* species contain anti-inflammatory, antidiabetic, and anticancer compounds, namely, flavonoids, tannins, phenolics, and alkaloids [129]. *Prosopis juliflora* Sw. DC. (mesquite) is an invasive species in India with reported ethnomedicinal uses, for the treatment of eye and digestive disorders, to name a few [130]. Mesquite extracts display relevant bioactivities, including anti-inflammatory, ascribed mainly to its content in alkaloids [130]. A leaf methanol extract from *Prosopis juliflora* Sw. DC. (mesquite) had cytotoxic effects on Molt-4 cells, with IC_50_ values of 90.5, 42.5, and 20.0 μg/mL after 24 h, 48 h, and 72 h of incubation, respectively, using mitomycin-C (6 µg/mL) as a positive control (Table 1). The extract was less toxic toward “normal” cells (mitogen stimulated T-lymphocyte cultures from peripheral human blood) [130]. A genotoxic assessment using a cytokinesis-block micronucleus assay reported that the number of micronuclei showed an increasing pattern with the application of increasing concentration of the extract [130].

The genus *Sesbania* comprises 60 to 85 species of herbs, shrubs, and trees distributed mostly in the tropical and subtropical regions of Africa, Asia, Australia, and America. *Sesbania* species have relevant pharmacological properties such as anti-inflammatory and antidiabetic [131,132,133]. *Sesbania grandiflora* (Akatti) (sesbania or agathi), is a small perennial tree with high levels of vitamins and minerals associated with anti-inflammatory, analgesic, and antipyretic properties [134,135], and the anticancer properties of its fruit are mentioned in the Ayurvedic literature [136]. Water, ethanol, and acetone leaf extracts of *S. grandiflora* reduced the viability of IMR32 and HT29 cells with IC_50_ values of 200 µg/mL after 24 h of incubation using doxorubicin as a control [137]. However, the chemical composition of the extract was not unraveled by the authors. Additional studies regarding the anticarcinogenic activity of other halophytes of this family are depicted in Table 1.

### 2.11. Juncaceae Family

*Juncaceae* currently contains eight genera and 522 species [138] of herbs adapted to salty marshes or badly drained soils and accumulate different phytoconstituents such as flavonoids, triterpenes, steroids, and phenolic acid derivatives [139,140]. A phenanthrene, juncunol (1,7-dimethyl-5-vinyl-9,10-dihydrophenanthren-2-ol), was identified in a diethyl ether extract of leaves of *Juncus acutus* (L.) Torr. ex Retz. (spiny rush) and displayed selective in vitro cytotoxicity toward HepG2, HeLa, and MDA-MB-468 cell lines [141]. Juncunol had an IC_50_ value of 18 µM in HepG2 cells after 72 h of incubation, determined using the MTT assay, and it induced an increase in the number of apoptotic cells in a concentration-dependent manner (IC_50_ value ± 25%) accompanied by a decrease in the Δψm [141,142]. Juncunol induced cell-cycle arrest in the G0/G1 phase, while showing no hemolytic properties. In silico studies indicate that that compound seems to bind between GC base pairs and, thus, may act as a DNA intercalator [142].

### 2.12. Malvaceae Family 

This family of flowering plants currently contains 243 genera and 5461 species [143]. A leaf decoction from *Thespesia populnea* L. Sol. ex Corrêa (portia tree) had a high content of total phenols and flavonoids and showed cytotoxic and antiproliferative properties toward HEP-2 cells, as observed in the MTT (IC_50_ = 120.02 µg/mL after 24 h of incubation) and SRB (IC_50_ = 77.06 µg/mL) assays [144]. Treated cells showed apoptotic characteristics including membrane blebbing, cell shrinkage, nuclear and cytoplasmic condensation, and formation of apoptotic bodies [144]. However, no attempt was made to identify the active molecules present in this sample. The chloroform-soluble fraction of a methanol bark extract of portia tree caused a reduction in cellular viability of MDA-MB-231 and MCF7 cells, with IC_50_ values of 23.97 and 20.62 µg/mL respectively, after 24 h of incubation [145]. Chemical analysis of the extract using GC–MS revealed the presence of steroids such as cis-androsterone and fatty-acid derivatives, which display cytotoxic activity against cancer cell lines [108,145].

### 2.13. Myrtaceae Family 

*Myrtaceae* presently includes 134 genera and 6614 species [146]. Focus is given to the genus *Eucalyptus*, especially *Eucalyptus camaldulensis* Dehnh, because is a salt-tolerant species with reported antitumoral properties. *Eucalyptus* spp. are mainly cultivated for timber and paper production, but the presence of bioactive compounds, such as triterpenoids, flavonoids, and tannins, confers *E. camaldulensis* with relevant bioactivities, including antitumoral [147]. Methanol, ethyl acetate, *n*-butanol, and water extracts from leaves of *E. camaldulensis* reduced the viability of MCF7 and MDA-MB-231 cell lines, according to the MTT and SRB assays [148]. The ethyl acetate extract had the highest cytotoxicity with IC_50_ values of 26.7 and 7.9 µg/mL for MTT and SRB in MCF7 cells, and IC_50_ values of 34.4 and 4.9 µg/mL for MTT and SRB in MDA-MB-231 cells after 24 h of incubation [148]. No attempt was reported by the authors to disclosure the molecules responsible for the detected cytotoxic activity.

### 2.14. Plantaginaceae Family

This family currently contains 101 genera and 2165 species [149], widely distributed worldwide but with preference for temperate zones [150]. *Bacopa monnieri* (L.) Wettst (water hyssop) is a wetland macrophyte and contains bacopaside (II) (saponine), with reported anticarcinogenic properties [151,152]. The anticarcinogenic properties of artificially digested (artificial saliva and artificial gastric juice) methanol extracts from *B. monnieri* reduced the motility (capacity of cell migration in a process of cancer development) of DU145 cells [153]. Nevertheless, the IC_50_ value was not determined by the authors. The extracts, analyzed by HPLC, were rich in phenolic compounds such as chlorogenic, caffeic, and syringic acids and bacoside A, with reported anticarcinogenic properties [154,155,156,157]. 

Within the Plantaginaceae family, the *Plantago* genus is the largest with 275 annual and perennial species widespread around the world, some with traditional uses as antipyretic, anti-inflammatory, and antitumoral agents [158]. *Plantago major* L. (fleawort) is an important medicinal plant due to its richness in bioactive metabolites, such as alkaloids and flavonoids, especially luteolin-7-O-β-glucoside, with antitumoral properties [159]. Silver particles produced by combining a water extract from *P. major* seeds and silver nitrate (AgNO_3_, 0.1 M) significantly decreased the viability of MCF7 cells with an IC_50_ value of approximately 12 µg/mL after 24 h of incubation [159]. However, no attempt was made by the authors to identify the bioactive compounds present in this extract. Information targeting other *Plantaginaceae* species is summarized in Table 1.

### 2.15. Plumbaginaceae Family

*Plumbaginaceae* encompasses several species adapted to survive under saline conditions and currently includes 1138 species distributed across 26 genera [160]. The genus *Plumbago* contains 18 species (shrubs or perennial herbs) characterized by the presence of flavonoids, phenols and saponins, conferring several properties including anticarcinogenic [161,162]. The genus *Limoniastrum* Heist. ex Fabr. comprises the halophytic species *Limoniastrum monopetalum* (L.) Boiss. (≡ *Statice monopetala* L.) and *Limoniastrum guyonianum* Boiss. [163,164], thriving in coastal and saline dry areas of the Mediterranean and northern Saharan Africa [165,166,167,168]. *Limoniastrum guyonianum* is traditionally used in the treatment of gastric infections [169]. A gall aqueous extract from *L. guyonianum* reduced the viability of HeLa cells with IC_50_ values of 170 and 140 μg/mL after 24 and 48 h of incubation, respectively [169]. This extract was rich in flavonoids and induced DNA hypomethylation and apoptosis due to its capacity to arrest cell-cycle progression in G2/M (Table 1). Due to the high levels of polyphenolic compounds detected in the extract, the antiproliferative and proapoptotic effects of such metabolites on HeLa cells were hypothesized by the researchers [169]. In another study, hexane, dichloromethane, ethanol, and methanol extracts from *L. densiflorum* (Guss.) Kuntze shoots were tested for cytotoxicity on cancer and “normal” cell lines using the resazurin reduction assay, after 48 h of treatment (Table 1) [170]. The dichloromethane extract was the most bioactive sample, with IC_50_ values of 29 and 85 μg/mL toward A-549 and DLD-1, without significantly reducing the viability of a human skin fibroblast cell line (Table 1). The major compounds present in that extracts were identified using RP-HPLC as *trans* 3-hydroxycinnamic acid, myricetin and isorhamnetin, which may be related to the observed cytotoxic activity [170].

### 2.16. Poaceae Family

This grass family currently includes 11,917 species divided across 796 genera [171,172]. In this family, only two genera of halophytes are reported with anticarcinogenic properties, namely, *Cynodon* and *Echinochloa*. The most representative *Cynodon* species is *Cynodon dactylon* L. Pers (Bermuda grass), a weed with several medicinal properties, such as antidiabetic, diuretic, and purifying, as well as in vitro cytotoxic properties [173]. The in vitro antitumoral properties of petroleum ether, dichloromethane, acetone, methanol/water (3/1), and water extracts from Bermuda grass were appraised on a breast cancer cell line, and cell viability was assessed by evaluating (3H)-hypoxanthine incorporation after 48 h of incubation [174] (Table 1). The highest cytotoxicity values were observed after application of the water, acetone, and petroleum ether extract, with IC_50_ values of 57.2, 38, and 39 μg/mL (Table 1) [174]. The acetone and ether petroleum extracts were profiled using LC–MS, allowing for the identification of several bioactive anthocyanins, namely, delphinidin, petunidin, malvidin, and cyanidin glucosides (delphinidin-3-O-acetylglucoside, petunidin-3-*O*-caffeoylglucoside-5-*O*-glucoside, petunidin-3-*O*-coumarylglucoside-5-*O*-glucoside, malvidin-3-*O*-monoglucoside, delphinidin-3-*O*-acetylglucoside-pyruvic acid, petunidin-3-*O*-acetylglucoside-5-*O*-glucoside, and cyanidin-3,5-*O*-diglucoside) [174] (Table 2). There is no information related to the specific anticancer properties of the detected anthocyanins; however, several others, such as delphinidin, cyanidin, malvidin, and corresponding glycosides, exhibit antitumoral properties in different cell lines [174]. An ethanol extract (70%) from grains of *Echinochloa crus-galli* (L.) P. Beauv. (cockspur grass) significantly reduced the viability of HCT-116 and HeLa cell lines, with IC_50_ values of 11.2 and 12.0 μg/mL, respectively [175] (Table 1). The active ethanolic crude extract was fractionated using *n*-hexane, chloroform, ethyl acetate, and *n*-butanol, with the ethyl acetate fraction exhibiting the lowest IC_50_ value (3.8 μg/mL). Fractionation of this active fraction allowed the isolation and identification of eight phenolic compounds, which exhibited significant cytotoxic activities toward the tested cells [175]. The most active molecules were 5,7-dihydroxy-3′,4′,5′-trimethoxy flavone and 5,7,4′ -trihydroxy-3′,5′-dimethoxy flavone (tricin), the latter with IC_50_ values of 7.2, 8.6, 10.8, and 19.9 μM against HepG2, HeLa, HCT116, and MCF7 cell lines, respectively, with similar results to the commercial anticancer drug, doxorubicin [175]. Further data on the antitumoral properties of other species of the *Poaceae* family can be found in Table 1.

### 2.17. Portulacaceae Family

This family contains one genus with presently 150 species [176] distributed worldwide, including the edible *Portulaca oleracea* L. (purslane), a halophyte succulent annual plant with important nutraceutical and antioxidant properties [177,178]. The anticarcinogenic activity of this species can be ascribed to the presence of several compounds, such as portulacerebroside A, portulacanones B, and 2,2′-dihydroxy-4′,6′-dimethoxychalcone [179] (Table 2). Portulacerebroside A induced apoptosis in HCCLM3 cells via activation of the p38 MAPK and JNK-triggered mitochondrial death pathway [180]. An ethanol extract from purslane seeds reduced the viability (MTT assay) of HepG2 cells in a concentration-dependent manner with IC_50_ values of approximately 75 µg/mL after 24 h of incubation via apoptosis induction according to cellular morphology modification, i.e., a reduction in cellular size and adhesion capacity (Table 1) [181]. Oil from seeds of the same species reduced the proliferation of HepG2 and A-549 cancer cell lines, at concentrations ranging from 250 to 1000 µg/mL as observed using the MTT and neutral red uptake assays, after 24 h of incubation in both cancer cell lines [182]. However, the high concentrations of the extract used (up to 1000 µg/mL) must be highlighted; even at 100 μg/mL, no reduction in cell viability was observed (Table 1) When treated with the highest concentration, cells exhibited morphological modifications typical of apoptotic cells, such as loss of cell adhesion capacity, shrinkage, and round shape [182]. However, the chemical profile of the extract was not determined by the authors.

### 2.18. Rhizophoraceae Family

This family includes mangroves and comprises currently 148 species and 15 genera [183,184]. It has two halophytic species with reported cytotoxic properties against human cancer cells, namely, *Bruguiera gymnorhiza* (L.) Lam (black mangrove) and *Ceriops tagal* (Pers.) C. B. Rob. (oriental mangrove) [185,186]. A methanol extract from black mangrove stem bark reduced the proliferation of HeLa, RAJI, and myeloma cell lines, with IC_50_ values of 133, 504, and 384 µg/mL after 24 h of incubation using doxorubicin as a standard control, via apoptosis induction, since DNA fragmentation was detected by fluorescence with double staining (ethidium bromide–acrydine orange) [187].

### 2.19. Solanaceae Family

*Solanaceae* contains 100 genera and 2925 species currently recognized [188], many of them with a high worldwide economic importance as cultivated crops, such as *Solanum lycopersicum* L. (tomato) [189]. The number of halophytic species with anticarcinogenic activity in this family is scarce, and reports have mainly focused on *Lycium barbarum* L. (Chinese wolfberry). Chinese wolfberry’s dried fruits are widely used in traditional Chinese medicine for its anticancer properties, potentially associated with the presence of different bioactive compounds, including polysaccharides (proteoglycans), scopoletin (phytoalexin), and 2-*O*-β-_D_-glucopyranosyl-_L_-ascorbic acid (vitamin C analog) [190]. A polysaccharide-rich aqueous extract from Chinese wolfberry fruits reduced the proliferation of SW480 and Caco-2 cell lines, as observed by the crystal violet and MTT assays, with IC_50_ values of 600 and 650 µg/mL respectively, after 4 days of incubation, with a high capacity to arrest cell-cycle progression in the G0/G1 phase [191]. An ethanol extract from the dried fruit residue of this species was cytotoxic toward A431 cells, with an IC_50_ value of 873.7 µg/mL after 24 h of incubation, determined by water-soluble tetrazolium-8-[2-(2-methoxy-4-nitrophenyl)-3-(4-nitrophenyl)-5-(2,4-disulfophenyl)-2Htetrazolium] monosodium salt (CCK-8) assay [192]. The same authors conducted in vivo studies in mice submitted to an intraperitoneal injection of 50 mg/kg of the crude extract, reporting a lower volume and weight of the formed tumor and a higher survival rate of tumor-bearing nude mice [192]. Several phenolic amides were isolated from an ethanol extract from the stem of *L. barbarum* and tested toward glioma cell lines, using the MTS method [180]. 4-*O*-Methylgrossamide and grossamide exhibited the highest capacity to decrease the viability of GSC-3# and GSC-12# cell lines, especially the latter compound, with IC_50_ values of 6.40 and 5.85 µg/mL respectively. [193]. The precise mechanism of action was not appraised for the isolated compounds; however, there are reports that phenolic amides can inhibit tumor necrosis factor-alpha (TNF-α) and nuclear factor-kappa B (NF-κB), which may be related to the detected cytotoxic effect [194].

### 2.20. Tamaricaceae Family

*Tamaricaceae* contains five genera with currently 110 species [195], widely distributed around the world, and several are halophytic [196,197]. Tamarix plants (salt cedar) contain several bioactive metabolites such as methylferulate and syringic acid with antineoplastic properties [198]. Syringic acid was isolated from a methanol extract of *Tamarix aucheriana* (Decne.) Baum aerial parts and was cytotoxic toward SW1116 and SW837 cells in a time- (24–72 h of incubation) and dose-dependent manner, with IC_50_ values of 0.95 and 1.2 mg/mL, respectively [199]. Cytotoxicity was related to an increase in the percentage of apoptotic cells, attributed to the antimitogenic effect of syringic acid [199]. Methylferulate was isolated from the same type of extract and significantly reduced the viability of SW1116 and SW837 cells, with IC_50_ values of 1.7 and 1.9 mM after 24 h of incubation, respectively, being responsible for cell-cycle arrest in the S and G2/M phases and apoptosis induction, together with a reduction in NF-κB DNA-binding activity [200].

*Tamarix gallica* L. is traditionally used as an expectorant, antidiarrheal, and laxative, potentially attributed to the presence of ellagic and gallic acids, anthocyanins, tannins, flavonones, isoflavonones, and resveratrol in its leaves [201]. A methanol extract from shoots, leaves, and flowers of *T. gallica* significantly reduced the viability of Caco-2 cells, related to a decrease in DNA biosynthesis, as observed using fluorescence microscopy after DAPI staining, while a reduction in the percentage of cells in the G0/G1 and S phases was detected using flow cytometry [202].

## 3. Conclusions and Future Perspectives

As stated in Section 1, we included in this review paper published work, which we considered relevant, related to the cytotoxic effects of halophytes. Such reports included results obtained with crude extracts, fractions, and pure compounds, using in vitro and in vivo assays, and they occasionally presented IC_50_ values, phytochemical profile of the active samples, and identification of active molecules, along with proposed mechanisms of action. There are some drawbacks of the identified reports. For example, several authors tested considerably high concentrations of the target extracts, up to 1000 mg/mL, in some cases. Moreover, the IC_50_ values were not reported in a high number of studies, which impairs a critical evaluation of the obtained results. Additionally, several reports failed to establish the chemical composition of the cytotoxic extracts and/or the identification of the molecules responsible for the detected activity. Lastly, information about the effect of the extracts on cell lines from nontumoral origin and, therefore, the selectivity of the extracts toward cancer cells was not always provided. According to the American National Cancer Institute NCI), only crude extracts/fractions displaying IC_50_ values lower than 30 μg/mL in the preliminary assay are considered promising and should be further explored as sources of antitumoral compounds. Therefore, we consider that extracts from the species and extracts included in Table 3 could be explored in detail targeting the isolation of anticancer leads, namely, *C. ambrosioides*, *S. fruticosa*, *A. millefolium, C. soldanella, C. rotundata, M. indicus, T. populnea, E. camaldulensis, P. major, P. lanceolata, E. crus-galli,* and *L. barbarum*. Moreover, isolated compounds with IC_50_ values ≤10 μM are considered promising anticancer leads. Therefore, we highlight the compounds in Table 3, namely, 6′-*O*-(*n*-butanol) ilekudinoside B ester (*A. marina)*, 5,7-dihydroxy-3′,4′,5′-trimethoxy flavone and tricin (*E. crus-galli*), Portulacerebroside A (*P. oleraceae*), and grossamide (*L. barbarum).*

**Table 1:** List of halophyte species, organs and extracts tested, cell lines/types of cancer assessed, and obtained half-maximal inhibitory concentrations (IC_50_) values.

Family	Species	Organs	Extract	Cell Line	IC_50_	Proposed Mechanism of Action	Ref.
*Acanthaceae*	*Acanthus ebracteatus* Vahl	Shoots	Protein hydrolysate	A431 cells (skin carcinoma)	425.9 ng/mL		[34]
	*Acanthus ilicifolius* L.	Leaves and roots	Ethyl acetate	MCF7 cells (breast carcinoma) PA1 cells (ovarian carcinoma)	24.22 μg/mL (leaf) 29.20 μg/mL (root); 15.74 μg/mL (leaf) 20.00 μg/mL (root)	nd	[33]
		Leaves	Ethanol	HepG2 cells (hepatocellular carcinoma)	100 μg/mL	Apoptosis induction (DNA damage)	[31]
		Roots	Water	HepG2 cells (hepatocellular carcinoma)	39.76 μg/mL	Apoptosis induction (DNA damage)	[32]
	*Avicennia alba* Blume	Leaves	Methanol	MCF7 and HeLa cells (breast and cervical carcinomas)	57.02 and 44.30 µg/mL	Reduction in cell size and cell detachment	[36]
		Leaves	Chloroform/methanol	WiDr cells (human colon carcinoma)	173.78 µg/mL	Apoptosis induction (cell arrest in the G0–G1 phase)	[37]
	*Avicennia marina* (Forssk.) Vierh	Leaves	Methanol	HeLa cells (cervical carcinoma)	107 µg/mL	nd	[40]
		Leaves	Methanol extract and fractions	MDA-MB-231 cells (breast carcinoma) HEK cells (human embryonic kidney)	MDA-MB-231 cells: Crude extract 250 µg/mL Active faction (luteonin) 28 µg/mL (97 µM)	Luteonin: apoptosis induction (DNA fragmentation, decreased expression of BCL2, decreased expression of TP53).	[203]
		Fruits	Isolated compounds from ethanol/butanol fractions of ethanol extract	GSC-3# and GSC-18# cells (human glioma stem cell lines)	6′-*O*-(*n*-butanol) ilekudinoside B ester: 12.21 µg/mL (14 µM) for GSC-3# and 5.23 µg/mL (6 µM) for GSC-18#	nd	[204]
		Leaves and stems	Ethyl acetate	MCF-7 cells (estrogen positive breast cancer)	na	Apoptosis induction (ROS production, disruption of Δψm), decrease in caspase-7 protein levels); autophagy	[39]
		Leaves and seeds	Water, ethanol, methanol, ethyl acetate extracts, and fractions from the latter	AU565, MDA-MB-231, and BT483 cells (breast cancer) HepG2 and Huh7 cells (liver) NIH3T3 cells (nontumoral)	Fraction 2-5: 0.75 μg/mL on AU565 cells Fraction 3-2-9: 2.1 μg/mL on AU565 cells	Apoptosis induction (DNA fragmentation, cell nucleus condensation and fragmentation, decreased PARP and caspase-8, increased caspase 3)	[38]
		Leaves	Ethyl acetate	Xenograft MDA-MB-231 tumor growth in nude mice	nd	Suppression of tumor growth	[38]
*Aizoaceae*	*Mesembryanthemum crystallinum* L.	Leaves	Ethanol extract, ethyl acetate and butanol fractions	HCT116 cells (colon carcinoma)	na	Ethyl acetate and butanol fractions: reduction of ROS, apoptosis induction). Butanol fraction: cell-cycle arrest at G2/M phase	[44]
	*Sesuvium portulacastrum* L.	Whole plant	Methanol, acetone, hexane, and diethyl-ether	MDA-MB-231 cells (breast carcinoma), IMR-32cells (neuroblastoma), HCT116 cells (colon carcinoma)	Hexane extract: 942.07, 703.40 and 407.87 µg/mL for MDA-MDB-231, IMR32 and HCT116 cells	Apoptosis induction: nuclear condensation, cell shrinkage, of apoptotic bodies	[45]
*Amaranthaceae*	*Arthrocnemum indicum* Willd. Moq.	Shoots	80% methanol	Caco-2 cells (colon carcinoma)	na	Decline of DNA synthesis, cell-cycle arrest at the G2/M phase.	[51]
	*Atriplex halimus* L.	Leaves	Ethanol	HepG2 cells (hepatocellular carcinoma) MCF-7 cells (human breast adenocarcinoma) A549 cells (lung cancer)	HepG2: 54.86 μg/mL MCF-7: 153.6 μg/mL A549: 101.9 μg/mL	Apoptosis induction (expression of TP53, BCL2, and BAX genes)	[54]
	*Chenopodium album* L.	Branches and leaves	Petroleum ether	A549 cells (lung carcinoma)	33.31 µg/mL	Cell-cycle arrest at the G1 phase	[63]
	*Chenopodium ambrosioides* L.	Whole plant	Essential oil	MCF7 cells (breast carcinoma)	18.75, 9.45 and 10.50 µg/mL after 6, 24 and 48 h of incubation	nd	[64]
	*Chenopodium ambrosioides* L.	Leaves	Essential oil, ethanol extract, dichloromethane fraction	RAJI cells (lymphoblast) K562 cells (lymphoblast)	Essential oil: 1.0 g/mL; dichloromethane fraction: 34.0 g/mL (RAJI cells) Ethanol extract: 47.0 g/mL (K562 cells)	nd	[65]
	*Chenopodium quinoa* Willd.	Seeds	Polysaccharide fraction	SMMC 7721 cells (liver cancer) MCF7 cells (breast carcinoma) L02 and MCF 10A cells (“normal” cell line)	121.4 μg/mL (24 h), 53.4 μg/mL (48 h) 83.5 μg/mL (24 h), 64.6 μg/mL (48 h)	nd	[66]
	*Salicornia brachiata* Roxb	Shoots	Methanol	HepG2 cells (hepatocellular carcinoma)	267.84 μg/mL	Modification of cellular morphology	[205]
	*Salicornia europaea* L.	Leaves	Ethyl acetate and methanol	MCF7 cells (breast carcinoma)	97.9 and 117.1 μg/mL	nd	[68]
	*Suaeda fruticosa* (L.) Forssk	Leaves	Hexane	HCT116 cells (colon carcinoma) HepG2 cells (hepatocellular carcinoma), and MCF7 cells (breast carcinoma)	17.2 μg/mL 33 μg/mL 28.1 μg/mL	Apoptosis induction, cell-cycle arrest at the G0–G1 phase, chromatin condensation, membrane blebbing	[74]
	*Suaeda fruticosa* (L.) Forssk	Shoots	Hexane, dichloromethane, methanol, and water	A549, DLD-1, Caco-2 and HT-29 cells (lung and colon carcinoma)	Hexane extract: 49, 10, 140 and 12 µg/mL	nd	[80]
	*Suaeda monoica* Forssk	Whole plant	Ethanol, methanol, acetone, and diethyl ether	MDA-MB-231 cells (breast carcinoma)	Ethanol: 172.38 µg/mL; methanol: 148.77 µg/mL; acetone: 185.56 µg/mL; diethyl ether: 60.18 µg/mL	nd	[206]
	*Suaeda palaestina* Eig Zohary	Shoots	Dichloromethane	A549 cells (lung carcinoma) HepG2 cells (hepatocellular carcinoma)	34.82 µg/mL 30.76 µg/mL	nd	[207]
	*Suaeda salsa* L.	Shoots	Acidic polysaccharide (molecular weight = 53.8 kDa; composition: mannose, rhamnose, glucuronic acid, galacturonic acid, galactose and xylose in a molar ratio of 0.6: 8.0: 1.0: 83.6: 5.0: 7.2).	MCF7 and MCF-10A cells (human breast carcinoma)	na	Apoptosis induction (reduction of Δψm, increase in the levels of BAX, cytochrome C, caspase-3 and caspase-9, decrease in the level of Bcl-2	[208]
*Apiaceae*	*Crithmum maritimum* L.	Whole plant	Hexane, ethyl acetate, methanol, ethanol	Huh7 and HepG2 cells (hepatocellular carcinoma)	na	Ethyl acetate extract: cell-cycle arrest at the G0/G1 phase after 24 h of incubation and in the G2/M phase after 48 h; increase in necrotic and apoptotic cells	[85]
	*Eryngium maritimum* L.	Shoots and roots	Water	HepG2 cells (human hepatocellular carcinoma) Hep2 cells (human laryngeal epidermoid U138-MG cells (human glioma) Vero cells (African green monkey kidney)	Shoots: 32.4 µg/mL; roots: 35.0 µg/mL Shoots: 50 µg/mL; roots: 30.3 µg/mL Shoots: 32.9 µg/mL; roots: 16.3 µg/mL	nd	[70]
*Asteraceae*	*Achillea millefolium* L.	Shoots	Methanol extract combined with bleomycin	DU145 cells (prostate carcinoma) and HFFF2 cells (human non-malignant fibroblasts)	nd	nd	[88]
		Aerial parts	Petroleum ether, ethyl acetate, methanol, water	K562 cells (human myelogenous leukemia), HeLa cells (human cervical carcinoma) MCF7 cells (human breast carcinoma) A549 cells (human non-small cell lung)	Ethyl acetate: 0.58 µg/mL (HeLa), 0.73 µg/mL (K562) Water: 0.87 µg/mL (MCF-7) Petroleum ether: 0.87 µg/mL (K562)	Ethyl acetate extract: pre-G1 apoptosis and cell growth arrest in G2/M (HeLa)	[90]
	*Limbarda crithmoides* (L.) Dumort	Aerial parts	Methanol extract, *n*-hexane, dichloromethane, and aqueous methanol-soluble fractions, isolated compounds	OCI-AML3 cells (acute myeloid leukaemia)	nd	10-acetoxy-9Z-chloro-8,9-dehydrothymol: apoptosis induction	[92]
*Brassicaceae*	*Cakile maritima* Scop.	Aerial organs	Methanol extract, and *n*-hexane, ethyl acetate, and methanol	Caco2 and HeLa cells (colon and cervical carcinoma)	Hexane fraction: 12 and 126 µg/mL	nd	[96]
*Convolvulaceae*	*Calystegia soldanella* L.	Whole plant	Methylene chloride and methanol extracts, *n*-hexane, 85% methanol, *n*-butanol and water fractions	HepG2 cells (hepatocellular carcinoma)	na	Methanol fraction: cell-cycle arrest at the G0–G1 and S phases, apoptosis induction	[100]
	*Calystegia soldanella* L.	Whole plant	Methanol	A549 cells (human lung cancer) Col2 cells (human colon cancer)	8.0 µg/mL 27.4 µg/mL	nd	[101]
	*Cressa cretica* L.	Shoots	Hydroalcoholic	HepG2 cells (hepatocellular carcinoma)	2300 µg/mL	Increased BAX, decreased BCL2	[102]
*Cymodoceaceae*	*Cymodocea rotundata* Ehrin.	Leaves	Silver particles produced by combining water extract and silver nitrate (AgNO_3_, 1 M)	MG63 cells (osteosarcoma)	25.31 µg/mL	nd	[105]
	*Cymodocea serrulata* (R. Br.) Aschers. & Magnus	Leaves	Silver particles produced by combining water extract and silver nitrate (AgNO_3_, 1 M)	A549 cells (lung carcinoma)	100 µg/mL	nd	[106]
		Shoots	Hydroethanolic	HepG2 cells (hepatocellular carcinoma)	82.92 µg/mL	nd	[107,108]
*Cyperaceae*	*Cyperus rotundus* L.	Rhizomes	Methanol, ethanol, and water.	MDA-MB-231 cells (breast carcinoma)	225 µg/mL	Apoptosis induction via upregulation of the death receptor 4 (DR4), DR5, and pro-apoptotic BAX, and downregulation of antiapoptotic BCL2	[112]
*Fabaceae*	*Alhagi maurorum Medik*	Aerial organs	Lupeol (isolated from a methanol extract)	MCF7, MDA-MB-231 and MCF 10A cells (breast carcinoma)	>100 µg/mL	Increased mRNA expression and level of TP53, caspase-3, and BAX genes, decrease in BCL2 gene expression	[118]
	*Glycyrrhiza glabra* L.	Root	Methanol	A549 cells (human lung carcinoma) HepG2 cells (human hepatocellular carcinoma) HaCaT cells (immortal human keratinocyte)	189.1–238 µg/mL 248.5 µg/mL 158.8–241.9 µg/mL	nd	[122]
		Root	Ethanol	HT-29 cells (colon carcinoma)	na	Downregulation of heat-shock protein 90 (HS90) gene expression	[123]
	*Glycyrrhiza uralensis* Fisch	Root	Ethanol/water (7:3, *v*/*v*)	HeLa cells (cervical carcinoma)	na	nd	[125]
	*Melilotus indicus* L.	Aerial parts	Methanol	HepG2 cells (human hepatocellular carcinoma) SNU-182 cells (hepatocellular carcinoma) L-02 cells (human “normal” hepatic)	16.60 µg/mL 13.21 µg/mL 90.9 µg/mL	Increase in the number of apoptotic cells, loss of mitochondrial membrane potential (Δψm)	[128]
	*Prosopis cineraria* L. Druce	Leaves	Methanol	MCF7 cells (breast carcinoma) and HBL-100 cells (noncancerous breast)	na	nd	[209]
	*Prosopis juliflora* Sw. DC.	Leaves	Methanol	Molt-4 cells (human T-cell leukemia)	90.5, 42.5 and 20.0 μg/mL (24 h, 48 h and 72 h of incubation)	Increased number of micronuclei	[130]
	*Sesbania grandiflora* L.	Leaves	Water, ethanol, and acetone	IMR32 and HT-29 cell lines (neuroblastoma and colon carcinoma)	200 µg/mL		[137]
*Juncaceae*	*Juncus acutus* L. Torr. Ex Retz.	Shoots	Diethyl ether, chloroform, methanol, and water	HepG2 cells (hepatocellular carcinoma) S17 cells (murine non-tumoral)	Ether extract: 6.2 and 34 µg/mL Juncunol: 18 µM (HepG2 cells)	Juncunol: increased number of apoptotic cells, decrease in the Δψm, cell-cycle arrest in the G0/G1 phase, no hemolytic properties	[129,130]
*Malvaceae*	*Thespesia populnea* Sol. Ex Corrêa	Leaves	Decoction	HEP-2 cells (epidermoid carcinoma)	120.02 µg/mL	Apoptosis induction (membrane blebbing, cell shrinkage, nuclear and cytoplasmic condensation, apoptotic bodies)	[144]
		Stem bark	Methanol	MDA-MB-231 and MCF7 cells (breast carcinoma)	23.97 and 20.62 µg/mL	nd	[145]
*Myrtaceae*	*Eucalyptus camaldulensis* Dehnh	Leaves	Ethyl acetate	MDA-MB-231 and MCF7 cells (breast carcinoma)	26.7 and 34.4 µg/mL	nd	[148]
*Plantaginaceae*	*Bacopa monnieri* L. Wettst	In vitro cultures (shoots)	Methanol and artificial saliva and gastric juice extracts	DU145 cells (prostate cancer)	nd	nd	[153]
	*Plantago lanceolata* L.	Leaves	Ethanol	MCF7, AMJ13, MDAMB and CAL51 cells (breast carcinoma)	0.674, 0.726, 0.251 and 0.024 mg/mL	nd	[210]
	*Plantago major* L.	Seeds	Silver particles produced by combining water extract and silver nitrate (AgNO_3_, 0.1 M)	MCF7 cells (breast carcinoma)	12 µg/mL	nd	[159]
*Plumbaginaceae*	*Limoniastrum densiflorum* (Guss.) Kuntze	Shoots	Hexane, dichloromethane, ethanol, and methanol	A-549 cells (human lung adenocarcinoma DLD-1 cells (human colon carcinoma) WS-1 cells (human skin fibroblasts)	Dichloromethane extract: 85 µg/mL (DLD-1)-and 29 µg/mL (A-549)	nd	[170]
	*Limoniastrum guyonianum* Boiss	Gall	Water	HeLa cells (cervical carcinoma)	170 µg/mL	DNA hypomethylation and apoptosis, cell-cycle arrest at G2/M, upregulation of p16INK4A, upregulation of UHRF1 and DNMT1	[169]
*Poaceae*	*Cynodon dactylon* L.	Whole plant	Petroleum ether, dichloromethane, acetone, methanol/water (3/1) and water	MCF7 cells (breast carcinoma)	Water extract: 57.21 μg/mL; acetone extract: 38 μg/mL; petroleum ether extract: 39 μg/mL	nd	[174]
		Whole plant	Petroleum ether	HEP-2, HeLa, and MCF7 cells (laryngeal, cervical, and breast carcinoma)	0.20, 0.62 and 1.02 mg/mL	Apoptosis induction (DNA fragmentation)	[211]
	*Echinochloa crus-galli* L.	Grains	Ethanol extract, *n*-hexane, chloroform, ethyl acetate, *n*-butanol fractions, isolated compounds	MCF7, HCT116, HeLa, and HepG2 cells (breast, colon, cervical, and liver carcinoma)	Crude extract: 12.0, 11.2, 18.9, and 14.2 μg/mL Ethyl acetate fraction: 3.8 μg/mL	nd	[175]
*Portulacaceae*	*Portulaca oleracea* L.	Seeds	Ethanol	HepG2 cells (hepatocellular carcinoma)	75 µg/mL	Round cells with reduced size and adhesion	[181]
		Seeds	Oil extraction	HepG2 and A549 cells (liver and lung carcinoma)	nd	Loss of cell adhesion capacity, shrinkage, round shape	[182]
*Rhizoporaceae*	*Bruguiera gymnorhiza* L. Lam	Stem bark	Methanol	HeLa cells (cervical cancer), Raji cells (lymphoma) and myeloma cells (leukemia)	133, 504, and 384 µg/mL	Apoptosis induction (DNA fragmentation)	[187]
*Solanaceae*	*Lycium barbarum* L.	Fruits	Water	SW480 and Caco-2 cells (colon carcinoma)	600 and 650 µg/mL	Cell-cycle arrest at G0/G1	[191]
		Fruits	Ethanol	A431 cells (cutaneous squamous cell carcinoma)	873.7 µg/mL	Reduced expression of Ki67 and PCNA proteins, increased expression of caspase-3, reduction of BCL2, downregulation of LC3II, reduced the phosphorylation of ERK1/2, and upregulation of JNK. Reversion of the regulation of Beclin1, LC3II, Bcl-2, and cl-caspase-3	[192]
		Fruits	Ethanol	BALB/c nude mice transplanted tumor model established by subcutaneous injection of A431 cells	nd	Increased survival rate, reduced tumor volume and weight, downregulation of Ki67 and MMP-2	
*Tamaricaceae*	*Tamarix aucheriana* (Decne.) Baum	Shoots	Syringic acid isolated from a methanol extract	SW1116 and SW837 cells (colorectal carcinoma)	0.95 and 1.2 mg/mL	Increased percentage of apoptotic cells	[199]
		Shoots	Methyl ferulate isolated from methanol extract	SW1116 and SW837 cells (colorectal carcinoma)	1.7 and 1.9 mM	Cell-cycle arrest in the S and G2/M phases, apoptosis induction, reduction in NF-κB DNA-binding activity	[200]
	*Tamarix gallica* L.	Shoots, leaves and flowers	80% methanol	Caco-2 cells (colon carcinoma)	na	Decreased DNA synthesis, cell-cycle arrest at G2/M phase; modification in the levels of cyclin B1, p38, Erk1/2, Chk1, and Chk2	[202]

nd: not determined; Δψm: mitochondrial membrane potential, na: data not available.

**Table 2:** Identified molecules in active extracts from halophyte species.

Family	Species	Chemical Compounds	Chemical Structure	Class	Ref.
*Acanthaceae*	*Avicennia alba Blume*	Catechol borane	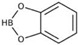	Organoboron	[36]
		Neophytadiene	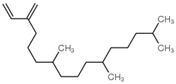	Terpenoid (diterpene)	[36]
		Hexadecanoic acid	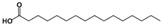	Fatty acid (saturated)	[36]
	*Avicennia marina* (Forssk.) Vierh	Luteonin	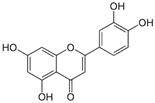	Phenolic (flavonoid)	[203]
*Amaranthaceae*	*Artrochnemum indicum*	Gallic acid	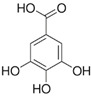	Phenolic (phenolic acid)	[51]
		Cyanidin	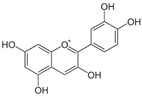	Phenolic (flavonoid)	[51]
		Chrysoeriol	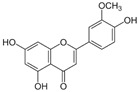	Phenolic (flavonoid)	[51]
		Quercetin	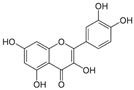	Phenolic (flavonoid)	[51]
		Catechol	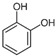	Phenolic (benzenediol)	[51]
		Luteolin	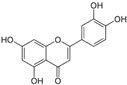	Phenolic (flavonoid)	[51]
	*Chenopodium ambrosioides* L.	Ascaridol	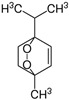	Monoterpene	[65]
*Asteraceae*	*Achillea millefolium*	Chlorogenic acid	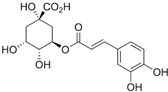	Phenolic (phenolic acid)	[90]
		*p*-Coumaric acid	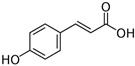	Phenolic (phenolic acid)	[90]
		Rosmarinic acid	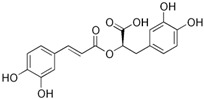	Phenolic (polyphenol)	[90]
		Apigenin	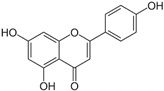	Phenolic (flavonoid)	[90]
	*Glycyrrhiza* sp.	Glycyrrhizin	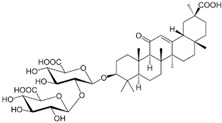	Saponin	[120,121]
	*Limbarda crithmoides* (L.) Dumort	10-acetoxy-8,9-epoxythymol tiglate	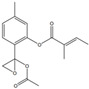	Thymol derivative	[92]
		10-acetoxy-9Z-chloro-8,9-dehydrothymol	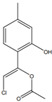	Thymol derivative	[92]
*Brassicaceae*	*Cakile maritima* Scop.	2-hydroxy-1,8-cineole	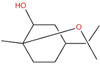	Monoterpenoid	[96]
		Decane	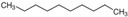	Alkane	[96]
		Limonene	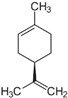	Monoterpenoid	[96]
*Fabaceae*	*Alhagi maurorum* Medik	Lupeol	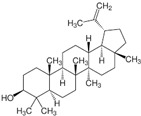	Terpenoid (triterpenoid)	[118]
	*Glycyrrhiza uralensis* Fisch	Isoquercitrin	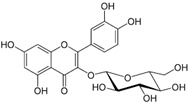	(Phenolic) flavonoid	[125]
		4′-Demethylpodophyllotoxin glucoside	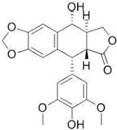	Lignan	[125]
		Podophyllotoxin	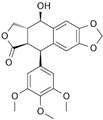	Lignin	[125]
*Juncaceae*	*Juncus acutus* L.	Juncunol	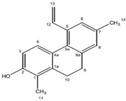	Phenanthrene	[141]
*Plantaginaceae*	*Bacopa monnieri* (L.) Wettst	Bacopaside (II)	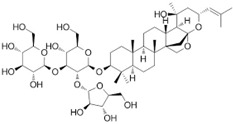	Terpenoid (isoprenoid)	[153]
	*Plantago lanceolata* L.	*O*-Coumaric acid	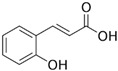	Phenolic (phenolic acid)	[210]
		Rutin	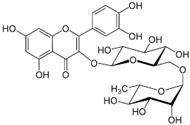	Phenolic (flavonoid)	[210]
		Myricetin	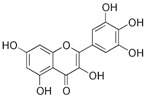	Phenolic (flavonoid)	[210]
		Quercetin	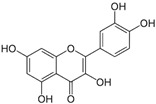	Phenolic (flavonoid)	[210]
		Kaempferol	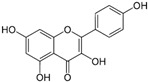	Phenolic (flavonoid)	[210]
*Plumbaginaceae*	*Limonium densiflorum* (Guss.) Kuntze	*trans* 3-Hydroxycinnamic acid	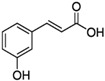	Phenolic (phenolic acid)	[170]
		Myricetin	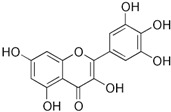	Flavonoid (phenolic)	[170]
		Isorhamnetin	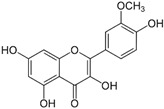	Flavonoid (phenolic)	[170]
*Poaceae*	*Cynodon dactylon* L. Pers	Delphinidin-3-*O*-acetylglucoside	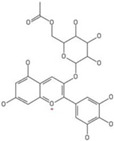	Anthocyanin	[174]
	*Echinochloa crus-galli*	5,7-Dihydroxy-3′,4′,5′-trimethoxy flavone	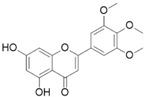	Phenolic (flavonoid)	[175]
		5,7,4′-Trihydroxy-3′,5′-dimethoxy flavone (tricin)	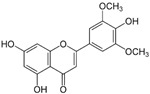	Phenolic (flavonoid)	[175]
*Portulacaceae*	*Portulaca oleracea* L.	Portulacerebroside A	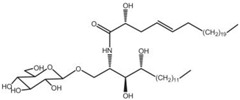	Cerebroside	[179,180]
		Portulacanones B	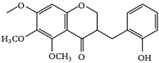	Phenolic (flavonoid)	[179,180]
		Portulacanones C	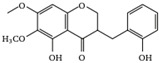	Phenolic (flavonoid)	[179,180]
		Portulacanones D	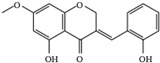	Phenolic (flavonoid)	[179,180]
		2,2′-Dihydroxy-4′,6′-dimethoxychalcone	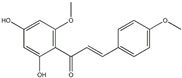	Phenolic (flavonoid)	[179,180]
*Solanaceae*	*Lycium barbarum* L.	4-*O*-Methylgrossamide	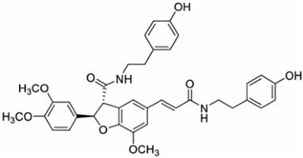	Phenolic amide	[193]
		Grossamide	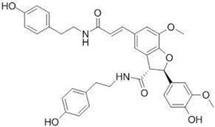	Phenolic amide	[193]
	*Lycium barbarum* L.	Scopoletin	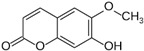	Phenolic (coumarin)	[190]
		2-*O*-*β*-_D_-Glucopyranosyl-L-ascorbic acid	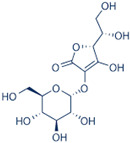	Glucoside	[190]
*Tamaricaceae*	*Tamarix aucheriana* (Decne.) Baum	Syringic acid	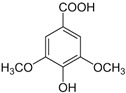	Phenolic (phenolic acid)	[198,200]
		Methyl ferulate	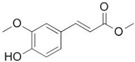	Phenolic (phenolic acid)	[198,200]

**Table 3:** Cytotoxic activity of selected halophytes species, including types of extracts, compounds, cell lines tested, obtained IC_50_ values, and proposed mechanisms of action. The criterion of cytotoxic activity for the crude extracts, as established by the American National Cancer Institute (NCI), was considered as an IC_50_ < 30 μg/mL upon 48 h or 72 h of incubation [24].

Family	Species	Organ	Extract/Fraction	Compound Detected in the Extract	Cell lines	IC_50_ Values	Mechanism	Ref.
*Amaranthaceae*	*Chenopodium* *ambrosioides* L.	Whole plant	Essential oil	nd	MCF7	18.75, 9.45 and 10.50 μg/mL at 6, 24 and 48 h	DNA fragmentation	[64]
	*Suaeda fruticosa* (L.) Forssk.	Leaves	Hexane	Monoterpenes (dihydrojasmone, jasmolone, terpinene-4-ol), diterpenes (pimaric acid, steviol, and momilactone B) and phenolics (quercinol, zingerone, zingerol, neovaflan)	HCT116	17.2 µg/mL	Cell-cycle arrest at the C0–G1 phase and apoptosis induction	[74]
	*Suaeda fruticosa* (L.) Forssk.	Shoots	Dichloromethane	nd	DLD-1 HT-29	10 µg/mL 12 µg/mL	nd	[80]
*Asteraceae*	*Achillea millefolium* L.	Aerial parts	Ethyl acetate, water, and petroleum ether extracts	Ethyl acetate: p-coumaric acid, chlorogenic acid, and apigenin Water extract: rosmarinic acid	K562 cells HeLa cells MCF7 A549 cells	Ethyl acetate: 0.58 µg/mL (HeLa), 0.73 µg/mL (K562) Water: 0.87 µg/mL (MCF-7) Petroleum ether: 0.87 µg/mL (K562)	Ethyl acetate extract: preG1 apoptosis and cell growth arrest in G2/M (HeLa)	[90]
*Brassicaceae*	*Cakile maritima* Scop.	Aerial parts	Hexane	GC-MS, decane, limonene, nonaldehyde, dodecane	CaCo2	12 μg/mL	nd	[96]
*Convolvulaceae*	*Calystegia soldanella* (L.) R.Br. ex Roem. & Schult.	Whole plant	85% aqueous methanol fraction of crude methanol extract	nd	HepG2	<30 μg/mL	Cell-cycle arrest at the G0–G1 and S phases, apoptosis induction	[100]
		Whole plant	Methanol	nd	A549 cells (human lung cancer) Col2 cells (human colon cancer)	8.0 µg/mL 27.4 µg/mL	nd	[101]
*Cymodoceae*	*Cymodocea rotundata* EhrenbHempr. ex Aschers.	Leaves	Water	nd	MG63	25.31 µg/mL	nd	105
*Fabaceae*	*Melilotus indicus* L. All.	Aerial parts	Methanol	nd	HepG2 SNU-182	16.6 µg/mL 13.21 µg/mL	Increase in the number of apoptotic cells, loss of Δψm.	[128]
	*Prosopis juliflora* Sw. DC.	Leaves	Methanol	nd	Molt-4	20.0 μg/mL after 72 h	Reduction in micronuclei or cell proliferation	[130]
*Malvaceae*	*Thespesia populnea* L. Sol. ex Corrêa	Bark	Chloroform fraction of methanol extract	Flavonoids, triterpenes, and tannins	MDA-MB-231 MCF7	23.97 µg/mL 20.62 µg/mL	nd	[145]
*Myrtaceae*	*Eucalyptus camaldulensis*	Leaves	Methanol, ethyl acetate, *n*-butanol, and water	nd	MCF7 MDA-MB-231	26.7 µg/mL 7.9 µg/mL 4.9 µg/mL	nd	[148]
*Plantaginaceae*	*Plantago major* L.	Seeds	Water	nd	MCF7	12 µg/mL	nd	[159]
	*Plantago lanceolata* L.	Leaves	Ethanol	Flavonoid glycosides: *O*-cumaric, rutin, myricetin, quercetin and kaempferol	CAL51	24 µg/mL	Apoptosis induction increase in the nuclei condensation	[210]
*Poaceae*	*Echinochloa crus-galli* (L.) P. Beauv.	Grains	70% ethanol	nd	MCF7 HCT116 HeLa HepG2	12.0 μg/mL 11.2 μg/mL 18.9 μg/mL 4.2 μg/mL	nd	[175]
		Grains		5,7,4-Trihydroxy-3,5-dimethoxy flavone (tricin)	MCF7 HCT116 HeLa HePG2	4.3 μg/mL 4.5 μg/mL 4.5 μg/mL 4.2 μg/mL	nd	[175]
*Solanaceae*	*Lycium barbarum* L.	Stems	Ethanol	Phenolic amides	GSC-3# GSC-12#	28 µg/mL 20 µg/mL	nd	[193]

nd: not determined; Δψm: mitochondrial membrane potential.

**Table 4:** Cytotoxic activity of selected isolated compounds from halophytes species, including cell lines tested, obtained IC_50_ values, and proposed mechanisms of action. The criterion of cytotoxic activity for the isolated compounds was an IC_50_ value ≤10 μM, according to the National Institutes of Health (NIH) for screening the NCI60 program [25].

Family	Species	Organ	Compound	Cell Lines	IC_50_ Values	Mechanism	Ref.
*Acanthaceae*	*Avicennia marina* (Forssk.) Vierh	Fruits	6′-*O*-(*n*-butanol) ilekudinoside B ester	GSC-18# (human glioma stem cell lines)	6 µM	nd	[204]
*Poaceae*	*Echinochloa. crus-galli* (L.) P. Beauv.	Grains	5,7-dihydroxy-3′,4′,5′-trimethoxy flavone	HeLa HePG_2_	3.0 µM 3.0 µM	nd	[175]
			5,7,4-trihydroxy-3,5-dimethoxy flavone (tricin)	HCT-116 HeLa HePG_2_	10.8 µM 8.6 µM 7.2 µM	nd	
*Portulacaceae*	*Portulaca oleracea* L.	Aerial organs	Portulacerebroside A	HCCLM3	<3.5 µM	Apoptosis induction via activation of the p38 MAPK and JNK-triggered mitochondrial death pathway	[180]
*Solanaceae*	*Lycium barbarum* L.	Stems	Grossamide	GSC-3# GSC-12#	10.2 µM 9.3 µM	nd	[193]

nd: not determined.
